# Optimal Magnetic Sensor Vests for Cardiac Source Imaging

**DOI:** 10.3390/s16060754

**Published:** 2016-05-24

**Authors:** Stephan Lau, Bojana Petković, Jens Haueisen

**Affiliations:** 1 Institute of Biomedical Engineering and Informatics, Ilmenau University of Technology, P.O. Box 100565, D-98684 Ilmenau, Germany; bojana.petkovic@tu-ilmenau.de (B.P.); jens.haueisen@tu-ilmenau.de (J.H.); 2 Biomagnetic Center, Department of Neurology, Jena University Hospital, Erlanger Allee 101, D-07747 Jena, Germany; 3 NeuroEngineering Laboratory, Department of Electrical and Electronic Engineering, The University of Melbourne, 3010 Parkville, Australia

**Keywords:** magnetocardiography (MCG), wearable multi-sensor systems, particle swarm optimization, source analysis, heart, cardiovascular diseases, inverse problems, matrix condition, magnetostatics, boundary element method

## Abstract

Magnetocardiography (MCG) non-invasively provides functional information about the heart. New room-temperature magnetic field sensors, specifically magnetoresistive and optically pumped magnetometers, have reached sensitivities in the ultra-low range of cardiac fields while allowing for free placement around the human torso. Our aim is to optimize positions and orientations of such magnetic sensors in a vest-like arrangement for robust reconstruction of the electric current distributions in the heart. We optimized a set of 32 sensors on the surface of a torso model with respect to a 13-dipole cardiac source model under noise-free conditions. The reconstruction robustness was estimated by the condition of the lead field matrix. Optimization improved the condition of the lead field matrix by approximately two orders of magnitude compared to a regular array at the front of the torso. Optimized setups exhibited distributions of sensors over the whole torso with denser sampling above the heart at the front and back of the torso. Sensors close to the heart were arranged predominantly tangential to the body surface. The optimized sensor setup could facilitate the definition of a standard for sensor placement in MCG and the development of a wearable MCG vest for clinical diagnostics.

## 1. Introduction

Magnetocardiography (MCG) non-invasively provides information about the electrical activity of the heart. During every heartbeat, a wave of excitation travels through the heart and causes the heart muscles to contract in spatio-temporal pattern. The ultra-low magnetic fields caused by the muscle contraction can be measured outside the body. Typical peak values of the magnetic induction B for MCG are of the order of 50 pT [1,2] and cardiac signals are in a frequency range of approximately 1–40 Hz. Pathological deviations of the heart contraction are reflected in the spatio-temporal MCG signals and can be used for diagnostics.

A key challenge in the design of a multi-sensor system for MCG is that a tailor-made geometry around the human torso is required that robustly samples the magnetic field patterns of all stages of the heart excitation. The sensor system should be wearable to allow for measurement of the heart’s response to physical exercise and normal daily activity. Consequently, the number of sensors will be restricted to reduce weight and energy consumption.

In the past, superconducting quantum interference devices (SQUIDs) have been used for MCG. However, these magnetic field sensors require cooling with liquid helium. Consequently, typical arrangements of sensors have been restricted to the inside of a stationary cryostat device [3] and sampled the human torso from only one side. Recent developments of non-superconducting magnetic sensor technology made it possible to measure the magnetic field at room temperature. Magnetoresistive (MR) sensor technologies [4,5,6,7,8] as well as optically pumped magnetometer technologies [9,10,11,12,13] have reached sensitivities that are suitable for MCG and at the same time allow for customized and flexible sensor arrangements.

Magnetoresistive materials change their resistance in relation to an external magnetic field and when utilized in a sensor design can provide very high sensitivities of 5146%/mT [14]. Their detectivity is limited primarily by 1/f noise [8,15]. Tunnel junction MR sensors, for example, can have 1/f noise of 47 pT/Hz^1/2^ at 1 Hz [14]. Currently, active research aims to reduce this noise with: (1) new materials, sensor designs and fabrication methods [16,17]; (2) combining series of sensor elements [6,18]; and (3) using microelectromechanical flux guides [18,19,20,21,22,23,24]. Flux guides concentrate the flux at the position of the sensor by factors of up to 100 [19]. The flux guides are made to mechanically oscillate at frequencies above 10 kHz [15]. This modulation shifts the operating frequency of the sensor into a band, where the 1/f noise is low. Given the prototypes and simulations, the detectivity is expected to reach a few pT/Hz^1/2^ at 1 Hz at room temperature [8,19,21]. Available MR sensors are already capable of detecting a heartbeat [4,6,7,18,23]. The signal average of a series of heartbeats can increase the signal-to-noise ratio (SNR) such that temporal features of the heartbeat of a patient are resolved. The single heartbeat will become accessible through the sensors that are currently in development. Chip-scale MR sensors are well suited for a magnetic sensor vest, because they are less than or around 1 cm in diameter [8,18,23,25], require only a few µW of power [8,14,17,25,26], are electrically safe with operating voltages around 1 V [14,20,25,26], emit little heat, and can be mass-produced at low cost [17].

A second sensor technology for a magnetic sensor vest is the optically pumped magnetometer (OPM). An OPM utilizes the Zeeman Effect using a cell filled with a heated gaseous alkali metal, such as cesium or potassium [9,10,13,27]. The vapor changes opacity in relation to an external magnetic field. This change is detected with a laser. OPMs can have detectivities of 5 fT/Hz^1/2^ at 1 Hz [28] and have been successfully used to measure cardiac fields [9,29,30,31,32,33]. While many experimental OPMs are 1–5 cm large to increase sensitivity [9,12,30,31,34,35], it is possible to miniaturize them to 5 mm diameter [28,29] and to reduce power consumption to approximately 5 mW [28]. Vapor cells operate at temperatures between 76 °C [12] and 200 °C [28] and need to be insulated from their environment, especially in a wearable vest device. A laser source and fiber-optical connections to each sensor are required. Current developments aim at increasing the dynamic range, developing vectorial OPMs [36], and integrating the OPM technology into multi-channel systems [27].

A complete sensor arrangement for measuring cardiac magnetic fields would consist of three-axial sensors lying on the faces of a box enclosing the whole torso [37]. This measurement setup is referred to as a golden standard. However, such a system cannot be realized in practice. In simulations, it was shown that arrangements measuring all three components of the magnetic field provide maximum information content [38,39,40]. Vectorial measurements can be achieved by combining uni-directional sensors. Examples are the modular system using 304 SQUID magnetometers [41] and the 195-channel Argos200 MCG system (Advanced Technologies Biomagnetics, Pescara, Italy) using sensor triplets.

Finely sampling the whole area around the heart requires too many sensors. Therefore, the number of sensors needs to be reduced without affecting the coverage of the whole heart volume and the reconstruction of current sources. In a simulation, Kang *et al.* [42] showed that regular arrangements of 3D sensors with larger inter-sensor distance can have a spatial resolving power and localization accuracy comparable to a dense regular arrangement of 3D sensors. In practice, however, a dense arrangement of single-component MCG sensors capturing the magnetic flux perpendicular to the chest wall has been established because of simpler production and lower costs.

Optimization of sensor arrangements to capture the heart excitation patterns was first implemented for electrocardiography (ECG). Lux *et al.* [43] collected body surface potential maps (BSPM) of 132 test persons and evaluated sub-selections of electrodes, concluding that 30–35 selected electrodes yield low enough error values for diagnostic purposes. A further principal component analysis of BSPMs [44] uncovered 12 principal components of the electric field captured by BSPMs. Barr *et al.* [45] found 24 out of 150 electrodes to be sufficient, whereas Finlay *et al.* [46] reported 32 electrodes as being sufficient. Kornreich *et al.* [47] added that the right sub-clavicular and left posterior axillary regions are diagnostically valuable. Dössel *et al.* [48] using a finite element simulation showed that a numerical optimization of electrode positions maximizing the condition of the lead field matrix produces comparable electrode arrangements. The ECG investigations concur in the finding that sets of diagnostically optimal electrodes can be found, but that there is no unique optimal arrangement [49].

More recently, magnetic sensor optimization for cardiac applications has been investigated with emphasis on numerical measures of how well a limited sensor array captures information about the cardiac current sources. Jazbinšek *et al.* [50,51] simulated and measured the magnetic field of the heart at the front and back of the torso. After sub-selection of sensors based on the root mean square error to the full sensor information they found 20 sensors in front of the heart, under the left arm and on the back of the torso. Nalbach and Dössel [52] used the condition number (CN) of the lead field matrix as optimization criterion. They simulated radial magnetometers around the torso in a tube shape and successively eliminated sensors that did not improve the CN. They found that the optimization of magnetic sensors can increase the reconstruction robustness. A limitation of all practical studies is that only magnetic sensors measuring the component radial to the body were considered.

In a previous study [53], we showed that the optimization of position and orientation of a set of single-component magnetic sensors on a plane in front of the torso can reduce the CN with respect to the 2-norm of the corresponding lead field matrix by one order of magnitude compared to a regular grid of sensors. We could reduce the number of sensors to approximately 20–30 without compromising the condition of the lead field matrix. Sensors tended to be placed in areas of strong magnetic field gradient.

The objective of this study is to optimize positions and orientations of a set of 32 magnetic sensors in a vest-like arrangement for robust reconstruction of the electric current distributions in the heart.

## 2. Materials and Methods

### 2.1. Dataset

**Figure 1:**
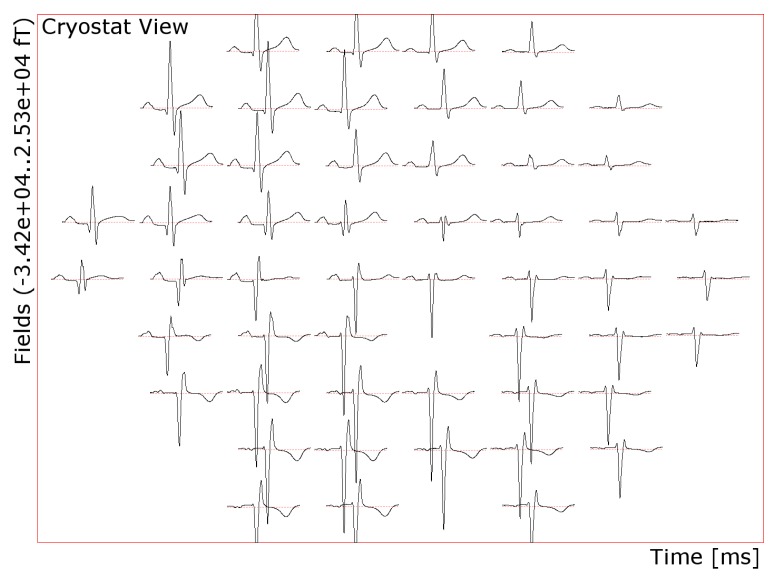
Position plot of the *B_Z_* component of the average heartbeat of the test person.

**Figure 2:**
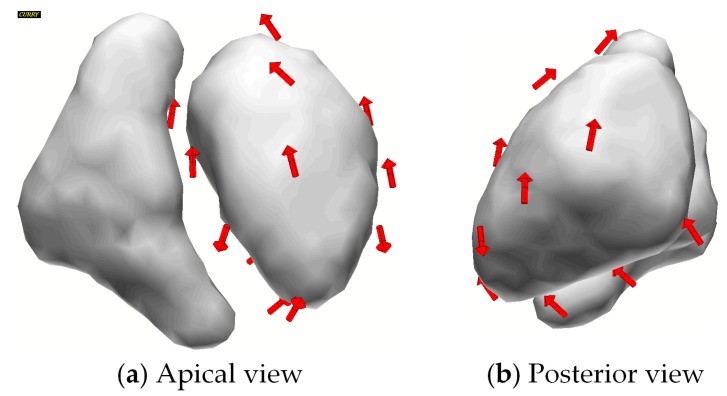
Source model of the cardiac field consisting of 13 dipoles around the left ventricle in: (**a**) apical view; and (**b**) posterior view.

Ethics approval (1448-11/04) was obtained from the Faculty of Medicine of the Friedrich-Schiller-University Jena. The magnetic field produced by the heartbeat (PQRST) of a 72-year-old cardiac patient was measured using a 195-channel vectorial MCG system (Argos200, Advanced Technologies Biomagnetics, Pescara, Italy) and averaged (*B_Z_* component is shown in Figure 1). A three-compartment boundary element model of the patient’s torso (conductivity 0.2 S/m) and lungs (0.04 S/m) was created from T1-weighted Magnetic Resonance Imaging. We modeled the cardiac sources with 13 dipoles [53] arranged regularly around the left ventricle, as shown in Figure 2. The orientation of the dipoles was fitted to the measurements with a minimum norm approach and L-curve regularization using the software Curry (NeuroScan Compumedics, Hamburg, Germany).

### 2.2. Goal Functions

Information on the geometry of the source space, the boundary element model and the sensor array is contained in the lead field matrix *L*

(1) $$\vec{b}=L\cdot \vec{d}$$

where $\vec{b}\in {ℜ}^{\#sensors}$ represents the magnetic flux density vector and $\vec{d}\in {ℜ}^{\#sources}$ represents the source dipole current density amplitudes. A well-conditioned *L* supports robust reconstruction. Therefore, our first measure of optimality of a sensor arrangement, or goal function, is the condition number with respect to the 2-norm, called *CN*, of *L* [54]

(2) $$CN(L)=\left\|L\right\|\cdot \left\|L^{+}\right\|=\frac{\sigma_{1}}{\sigma_{n}}$$

where ‖ ‖ denotes the 2-norm and *L*^+^ is the pseudo inverse of the matrix *L*. The condition number is equal to the ratio of the largest and the smallest singular value of the *L*. The second goal function is the *Skeel* condition number, proposed by Skeel [55] for square matrices and then generalized to rectangular matrices [56]. This measure of conditioning is defined as

(3) $$Skeel(L)=\left\|\left|L\right|\cdot \left|L^{+}\right|\right\|$$

where | | denotes that all elements of the matrices *L* and *L+* are replaced by their absolute values. The third goal function ρ, which was proposed recently by Eichardt *et al.* [57], is defined as

(4) $$\rho (L)=\sigma_{1}(L)/\left(\frac{1}{n}\sum_{i=1}^{n}\sigma_{i}(L)\right)$$

representing the ratio of the largest to the mean of singular values of *L*. This is in fact the inverse average decay of the singular values of *L*.

### 2.3. Optimization

Previous studies indicated that approximately 32 optimized sensors are sufficient to capture the complexity of the cardiac excitation expressed in the electric surface potential [43,45,46,50,51,52] and magnetic field [50,51,52,53]. Furthermore, 32 sensors are technically feasible in a vest design. Therefore, we optimized 32 sensor positions and orientations using a quasi-continuous constrained particle swarm optimization (PSO) approach [53,58], which is robust against local minima in the goal function.

**Figure 3:**
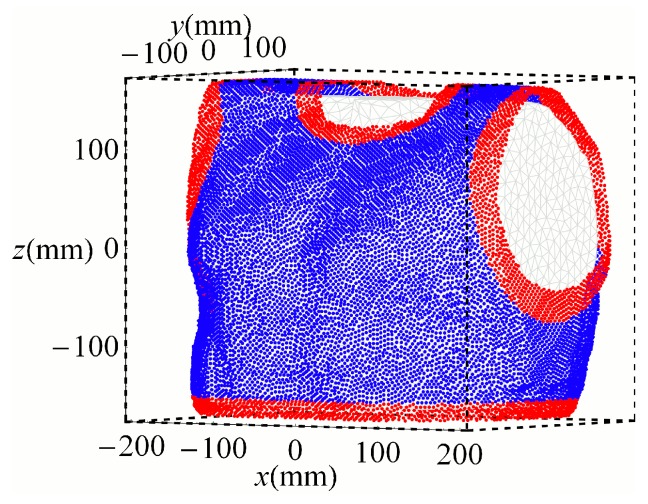
Search sensor space containing 19,759 positions on the vest; rejected sensor positions (red) and positions taken into analysis (blue).

Two optimization constraints were imposed. First, the sensor positions were restricted to the vest surface around the torso. Second, the sensors were modeled with a diameter of 1 cm, implying a minimum distance between sensors of 1 cm. The algorithm is described in [53] and implemented as an extension of the software toolbox SimBio [59]. The sensor search space consisted of the 19,759 nodes (Figure 3) of a triangulation (4 mm side length) of the dilated (14 mm) torso surface. The orientations were discretized to 30° steps yielding 62 different orientations per position.

The calculation of *L* in each iteration is computationally very expensive. Therefore, the sensor space was finely discretized and the *L* for all possible sensor positions and orientations was pre-computed. The matrix *L* for a particular sensor arrangement was then a subselection of rows of the pre-computed *L* with each row corresponding to one sensor.

Because the solution of this problem is not unique, we repeated the optimization 256 times using random initial sensor arrangements. This allowed us to sample the solution space and elicit generalizable patterns of optimal sensor arrangements for MCG.

### 2.4. Cluster Analysis

The sensor positions and orientations obtained from the 256 optimizations were combined into a set of 256 × 32 = 8192 sensors. A small fraction of sensors were positioned at the edges of the vest, indicating that some sensors would have been placed outside the vest surface and inside the body. Therefore, sensors positioned at the edge of the vest, *i.e.*, at the lower part of the torso and around the neck and around left and right arm as shown in red in Figure 3 were rejected. The remaining sensors were then partitioned into 32 clusters based only on their position using Partitioning Around Medoids (PAM) [60]. The PAM converged in less than 10 iterations. The medoid, as the most centrally located object of the cluster, was taken as the representative sensor position of the cluster.

The analysis of orientation vectors per cluster was done using the orientation matrix eigenvalue method [61]. The orientation vectors of MCG sensors belong to the group of axial data since the sensors pointing in exactly opposite directions measure the same magnitude of the magnetic flux density. Let the orientation vectors belonging to one cluster be the unit vectors placed in a unit sphere and let $\left(x_{1},y_{1},z_{1}),\ldots ,(x_{k},y_{k},z_{k}\right)$ be a collection of those unit vectors (axes). We define the orientation matrix as [61]

(5) $$T=\left(\begin{array}{ccc} \sum {x_{i}}^{2} & \sum x_{i}y_{i} & \sum x_{i}z_{i}\\ \sum x_{i}y_{i} & \sum {y_{i}}^{2} & \sum y_{i}z_{i}\\ \sum x_{i}z_{i} & \sum y_{i}z_{i} & \sum {z_{i}}^{2} \end{array}\right)$$

The variation of the moment of inertia gives the information about the scatter of the points on the unit sphere surface. The axis about which the moment is least is called the principal axis. Let $\tau_{1},\tau_{2},\tau_{3}$ be eigenvalues of the matrix *T*, $\tau_{1}\geq 0$, $\tau_{2}\geq 0$, $\tau_{3}\geq 0$, $\tau_{3}\geq \tau_{2}\geq \tau_{1}\geq 0$, and $u_{1},u_{2},u_{3}$ be corresponding eigenvectors. If $\tau_{1}=\tau_{2}=\tau_{3}=1/3$, then there is no axis with greater moment of inertia than any other; otherwise, $u_{3}$ is the principal axis. All clusters had a principal orientation axis and we took the respective axis as the representative orientation of the cluster. We quantified the shape of the orientation distribution for each cluster using the shape parameter $\gamma =\text{ln}(\tau_{3}/\tau_{2})/\text{ln}(\tau_{2}/\tau_{1})$ and the strength parameter $\xi =\text{ln}(\tau_{3}/\tau_{1})$ [61]. The shape parameter discriminates girdle-type distributions (γ < 1) from cluster-type distributions (γ > 1). The strength parameter measures the relative strength of the principal axis in a particular distribution. A uniform distribution corresponds to *ξ* = 0.

## 3. Results

### 3.1. Optimized Arrangements

The optimization reduced the goal functions *CN*, *Skeel* and ρ by factors of 11, 6, and 2, respectively, as shown in Table 1. Whereas the dispersion of goal function values was large in the set of random setups, it was small in the sets of optimized setups. Each individual optimized sensor arrangement showed a distribution of the sensors over the torso surface with more sensors above the heart, at the back of the torso close to the heart and under the left arm. The repeated optimization results showed similarities, but were not the same. A fraction of approximately 40%–47% sensors of each setup was placed at the edge of the sensor vest and excluded. The remaining sensors had 3220, 2814 and 3222 different locations covering 20.8%, 18.1% and 20.8% of locations of the discretized vest surface, for the *CN*, *Skeel* and ρ, respectively.

A representative setup was derived for each of the goal functions by clustering the sensors of the individual optimized setups. The goal function values of the three representative setups are shown in Table 2. The *CN*-based representative setup had a *CN* of 49, which is much closer to that of the individual optimized setups than the random ones. Similarly, its *Skeel* and ρ values (Table 2) were much closer to the respective optimal range than the random range (Table 1). The *Skeel*-based representative setup had a *Skeel* value (99) between the optimal range and the random range (Table 1). It performed non-optimal when evaluated with the *CN*, but comparatively well when evaluated with ρ (Table 2). The ρ-based representative setup had a ρ value much closer to the optimal range than the random range, but performed less favorable when evaluated with the *CN* and *Skeel*.

### 3.2. Clustered Positions

**Figure 4:**
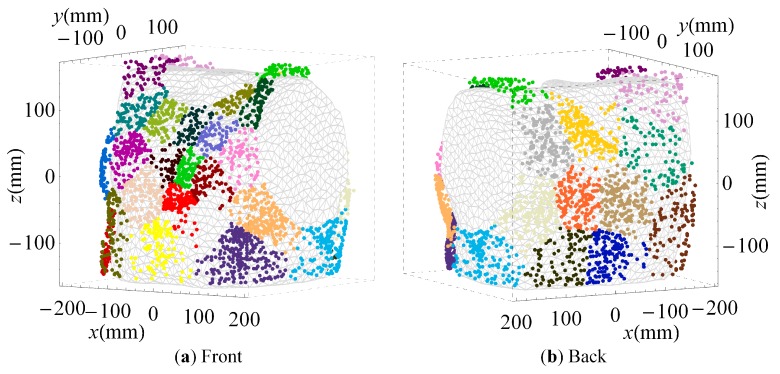
Color-coded sensor clusters for the 32 sensors array minimizing the *CN* displayed on the triangulated torso from the front (**a**) and from the back (**b**).

Color-coded sensor clusters based on the *CN* are displayed on the triangulated torso from the front and from the back in Figure 4. Most of the vest surface was utilized. Clusters on the front of the torso were smaller than clusters on the back. The smallest cluster size and the highest number of clusters were above the heart. These dense clusters were proximal to the heart’s atria or base and its apex (Figure 4a).

The median and maximal distance between sensor cluster medoids and single sensors were 30.8 mm and 108.1 mm, respectively. The most common distance to the respective medoid was approximately 27 mm, indicating an average equivalent cluster diameter of 2 × 27 mm = 54 mm.

**Figure A1:**
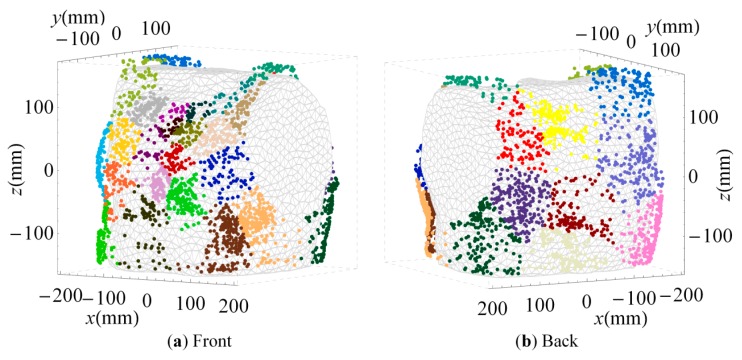
Color-coded sensor clusters for the 32 sensors array minimizing *Skeel* displayed on the triangulated torso from the front (**a**) and from the back (**b**).

The distribution of sensors using *Skeel* (Figure A1) was similar, but the coverage of the vest surface was sparser. When ρ was optimized (Figure A3), sensor clusters at the back of the torso close to the heart were smaller and closer to each other, than on sensor clusters at the front of the torso. The median and maximal distances between sensor cluster medoids and single sensors for *Skeel* and ρ were comparable to those using the *CN* (Table 1). The median distance to the cluster medoid was smallest for *Skeel*, whereas the maximal distance was smallest for the *CN*. The average equivalent cluster diameter for *Skeel* was 44 mm and for ρ was 24 mm.

### 3.3. Cluster Orientations

**Figure 5:**
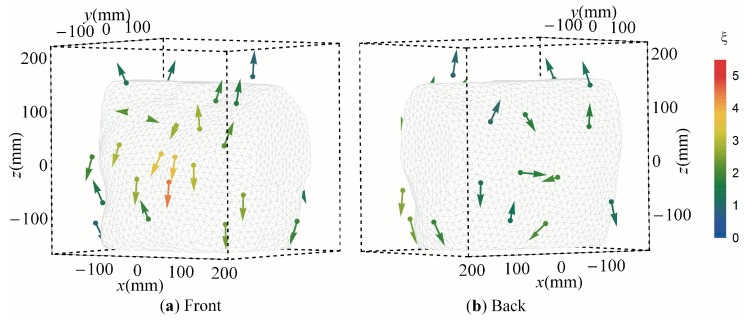
Optimized 32 sensors setup minimizing the *CN* from the front (**a**) and from the back (**b**). The dots represent the medoids and the arrows represent the principal axes of the orientation matrix. For best visibility, the arrow direction was selected to be the one that points away from the torso, rather than towards it. The color of each sensor representation indicates the strength parameter ξ.

Representative sensors of all the clusters based on *CN* are presented in Figure 5. The sensor orientations were mostly tangential to the body surface, especially above the heart at the front and back of the torso. However, at locations more distant to the heart, such as the shoulders, the sensors were oriented increasingly radial to the torso and heart.

The mean shape parameter of the orientation distributions per cluster was 1.76 (Table 1). This indicates more cluster than girdle-type orientation distributions. The large standard deviation of 1.8 indicates that there exist some clusters that are highly cluster-type and some that are highly girdle-type. The mean strength parameter of was 2.08. The strength of the principal axis of the orientation distribution is shown in Figure 5 as color-coding. It is the strongest (*ξ* = 3.5–4.5) in the frontal area above the heart, intermediate (*ξ* = 2–3) at the back close to the heart and weak (*ξ* = 1–2) at distant positions.

**Figure A2:**
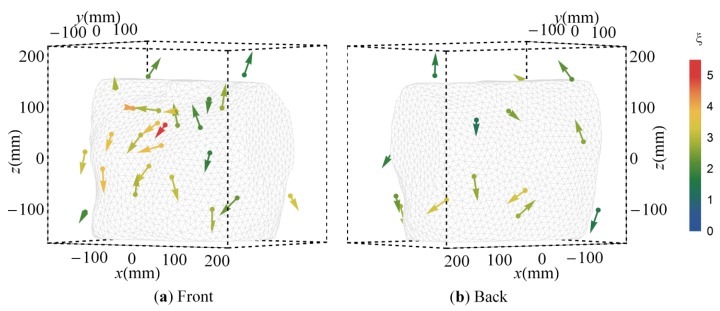
Optimized 32 sensor setup minimizing *Skeel* displayed on the triangulated torso from the front (**a**) and from the back (**b**). For best visibility, the arrow direction was selected to be the one that points away from the torso, rather than towards it. The color of each sensor representation indicates the strength parameter ξ.

**Figure A3:**
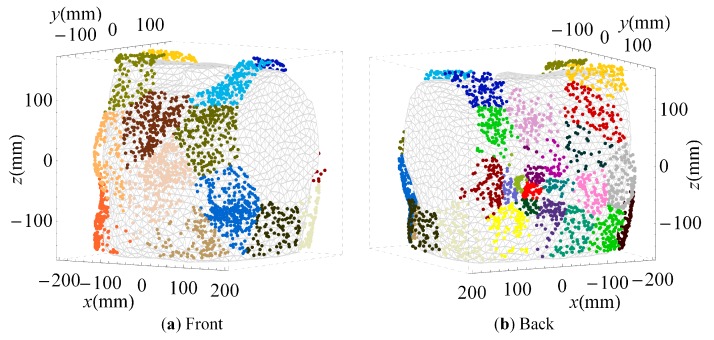
Color-coded sensor clusters for the 32 sensors array minimizing ρ displayed on the triangulated torso from the front (**a**) and from the back (**b**). For best visibility, the arrow direction was selected to be the one that points away from the torso, rather than towards it.

When *Skeel* was optimized (Figure A2), the orientation pattern of the representative sensors was similar to that of the *CN*. The densest sensor population at the front of the torso above the heart had the strongest principal axis. However, in contrast to the *CN* result, several sensors at the back of the torso had above average strength (*ξ* = 3.5–4). The average shape parameter of 1.45 was smaller than for the CN, indicating that the *Skeel* result was on average less cluster-shaped. The strengths of the principal axes of the orientation distributions were on average larger (mean *ξ* = 2.84) than for the *CN*.

**Figure A4:**
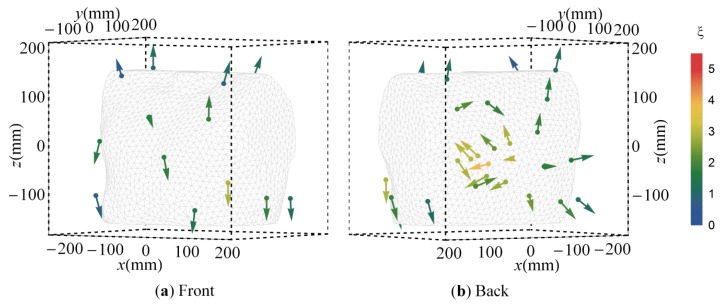
Optimized 32 sensor setup minimizing ρ displayed on the triangulated torso from the front (**a**) and from the back (**b**). For best visibility, the arrow direction was selected to be the one that points away from the torso, rather than towards it. The color of each sensor representation indicates the strength parameter ξ.

Using ρ (Figure A4), the orientations of the representative sensors were similar as well, but the dense set of sensors at the back of the torso near the heart had the highest degree of orientation conformity with *ξ* = 2.5–3.8. Several sensors at the right lower back reflected orientations radial to the torso with intermediate strength of *ξ* = 1.5–2.5. The mean shape parameter of the orientation distributions based on ρ was 1.91, which is higher than that of the *CN*. However, the mean strength parameter was 1.99, which is lower than that of the *CN* (Table 1).

## 4. Discussion

### 4.1. MCG Vest Optimization

The optimization of sensor positions and orientations in a vest-like setup reduced the condition number of *L* by approximately two orders of magnitude compared to that of a regular flat sensor array in front of the torso and approximately one order of magnitude compared to that of an optimized flat sensor array [53]. This indicates that optimized sampling of vectorial components of the cardiac magnetic field can greatly improve source reconstruction robustness. Previous studies using fixed radial sensor orientations [3,50,51,52] concur in the aspect that optimizing sensor positions around the torso can improve source reconstruction robustness.

Different sensor configurations performed equally well with respect to the condition of *L*, which is in agreement with Dössel [3], Nalbach and Dössel [52], Jazbinšek *et al.* [50,51], and ECG-based studies [43,45,46,47,49]. However, generalizable patterns could be established by clustering a large set of individual optimized sensor configurations. The clustering preserved commonalities across individual optimized setups, but not the optimized complementarity of the sensors within one individual setup.

A fraction of sensors was located at the edge of the vest. Reconstruction robustness was retained after rejecting these sensors, which confirms that these sensors were mostly redundant. In these noise-free simulations, 32 sensors were sufficient. This quantity is a comparable to previous MCG studies [50,51,52] and ECG studies [43,45,46].

### 4.2. Sensor Positions

Optimized setups exhibit non-uniform distributions of sensors over the whole torso. Multiple closely spaced sensors above the heart at the front of the torso are critical to capture the magnetic field of the cardiac sources at the shortest possible distance. Sensors at the back of the torso add valuable information, which improves the condition of the linear inverse problem. Earlier studies [50,51,52,62] using sensors with fixed radial orientation concur with this finding. Sato *et al.* [63], add that even the smaller atrial excitation can be observed with MCG sensors at the back of the torso. Sparser sampling of the remaining torso surface further improves the condition of the inverse problem by surrounding the torso. For example, our optimized setup includes sensors above the shoulders, which were not considered by earlier studies [50,51,52,62] presumably because this area is hard to reach with a cryostat-based system.

The necessary spatial sampling density varies with respect to the location on the body surface. For BSPM, Dössel [3] and Schneider *et al.* [64] reported necessary sensor distances from 10 mm at the apex to 100 mm at less informative locations. Our results for MCG show a similar but wider range with one representative sensor covering an average area of 54 mm diameter and a maximum area of less than 216 mm diameter. Based on sampling theory, Kim *et al.* [65] estimated that in a flat regular setup of purely tangential magnetometers in front of the torso the inter-sensor distance needs to be 40 mm or less to capture the theoretical spatial frequencies. This is in approximate agreement with our average distance of orientation-optimized sensors of approximately 54 mm.

The distance of neighboring sensors was larger than the assumed minimum distance of 1 cm, which was introduced as a constraint in the optimization. Consequently, a sensor footprint of 1 cm or less [8,18,23,28] should allow for sufficient spatial sampling density. It should also provide flexibility for placing two mono-directional sensors close to each other in order to sample separate vectorial components of the magnetic field close to one location.

Optimization with *Skeel* produced denser sampling of the front of the torso than of the back, which confirms the results based on *CN*. Optimization with ρ on the other hand produced a denser sampling of the back of the torso than of the front. The magnetic sensors on the back of the torso were mostly positioned on its left side, which was also suggested by Jazbinšek *et al.* [51]. The frontal region emphasized by *CN* and *Skeel* and the region on the back of the torso emphasized by ρ are both close to the heart compared to the rest of the torso. This allows for better sampling of higher spatial frequencies [64]. Both regions may contain corresponding localizing information and if so, then sampling one densely and the other more coarsely could be sufficient. However, complex pathologic excitation patterns could be more prominent on one side of the torso. Therefore, both regions should be sampled sufficiently densely for robust MCG.

The identified magnetic sensor positions are in principle agreement with electrode optimization studies [45,46,47,48,64] for ECG, which also found the areas above the heart at the front of the torso and at the back of the torso to be of diagnostic value. Further, ECG-based studies also suggest denser sampling above the heart at the front of the torso. ECG-electrodes are also placed close to the apex of the heart under the left arm, which is close to the main dipole axis during ventricular excitation. Whereas our optimized setup included several sensors in that area, there was no specifically dense sampling. This can be explained by the fact that at a BSPM maximum, indicating one end of a current dipole, the magnetic flux through a sensor is small.

Kornreich *et al.* [47] and Donnelly *et al.* [49] pointed out that diagnostically valuable electrode locations should be differentiated from high-amplitude ones. A lead is diagnostically valuable if a change of cardiac excitation pattern will result in a proportional, characteristic change of the signal detected by that lead. In the signal domain, this is equivalent to capturing topographic changes of the BSPM. In the source domain, this is equivalent to a well-conditioned relation between cardiac electric sources and sensor signals. In this context, our optimization of the condition of *L* can be clinically understood as an optimization of the combined diagnostic value of the sensor setup.

The vest setup allows us to sample all important sensor locations. This includes not only dense sampling at the front of the torso, but also its back as well as sparser sampling of the remaining torso surface. At the same time, the vest sensor space includes all possible sensor locations that are outside the body and close enough to the heart to be measurable. The coverage of the vest setup is, therefore, both necessary and sufficient for robust MCG.

### 4.3. Sensor Orientations

The sensor orientations correlate to the vectorial properties of the magnetic field at the respective sensor locations. For simplicity, consider a single equivalent dipole in the left ventricle pointing from the apex to the base of the heart during ventricular contraction (R-peak) (see Figure 2). The magnetic flux around this current exits and enters the torso in an arc shape (A good visualization is provided in Figure 6 of [66]). Our orientation-optimized sensors were arranged tangentially above the heart such as to sample the magnetic field between the exit and entry points. This indicates that such tangentially oriented sensors capture more information close to the cardiac source. The fewer radial sensors in our optimized setup were distant from the heart, e.g., at the shoulders. If we again consider for simplicity a single dipole in the heart, then these predominantly radial sensors are oriented to sample the field components pointing in approximately in that direction (see Figure 6 of [66]).

The value of tangential sensors is confirmed by Diekmann *et al.* [67] who showed in simulations and measurements that the SNR can be improved by a factor of up to 20 if the total field, including the tangential components above the heart, was considered rather than only radial components. Kandori *et al.* [68] based on measurements with their custom tangential sensor system further advocate the value of tangential sensor orientations. In line with our results, Kim and Lee *et al.* [65,69,70] point out that the tangential components of the MCG have a higher confidence of the source reconstruction of deep sources than radial components. A similar observation was reported by Hochwald and Nehorai [71] for the magnetoencephalography-based reconstruction of cortical sources in the center of a spherical magnetoencephalography sensor setup.

The analysis of orientation distributions showed that areas of the torso closest to the heart have the strongest principle axes. This indicates that at these positions the condition of the transformation between sources and sensor signals is orientation-sensitive and that specific beneficial orientations exist. The optimized setups based on the *CN* and *Skeel* were characterized by frontal sensors above the heart, whereas the optimized setup based on ρ favored sensors at the back close to the heart. Both sites may be to some degree alternative. Sensors distant from the heart, e.g., at the shoulders, appear to be beneficial, but less orientation specific.

In reality, the currents in the heart are more complex than a single dipole. In our source model we used 13 fitted dipoles to approximate the distributed nature of cardiac currents. The sensor setup was optimized to be sensitive to all 13 sources with their different orientations at the same time. Consequently, the optimized sensor setup reflects a combination of vectorial properties of all sources.

Previous sensor optimization studies [3,50,51,52] assumed fixed radial sensor orientations due to technical constraints of cryostat-based systems. This assumption influenced the resulting sensor arrangement. For example, Jazbinšek *et al.* [50,51] optimized a magnetic sensor setup by subselecting sensors from a dense array of radial (*B_z_*) sensors. Their selected radial sensors were in areas of medium to high *B_z_* amplitude, which are close to the torso exit and entry points of the magnetic field, as well as at local topographic *B_z_* flux density map features (see top row in Figure 3 of [50]). The availability of flexible room temperature sensor technology eliminates this technical constraint and allows for optimized vectorial sampling of the magnetic field for more robust reconstruction of cardiac sources from MCG signals.

### 4.4. Limitations and Future Work

A limitation of our study is the noise-free model. In practice, the low SNR will limit the utility of sensors distant to the sources in the heart. In future work, noise should be incorporated. This will increase the influence of signal strength on the selection of sensor positions and orientations and as a general tendency may promote positions closer to the heart.

For the diagnosis of the various functional pathologies of the heart, MCG recordings of a representative larger set of patients should be used as an input for modeling and optimization. Pathologic excitation patterns appear at specific time points during the heartbeat, e.g., the ventricular contraction during the ST segment. Future work could focus the source model on such indicative time points. In this context, the source model could be extended to include sources in the atria, the atrioventricular (AV) ring, the right ventricle, the sinus node, the AV node, and the nerve bundles [72,73,74].

As a next step, the optimized sensor setup should be validated by physically constructing it and performing measurements in control persons and patients. A practical challenge in this process will be to provide enough flexibility for different body shapes and movement, while maintaining accurate spatial co-registration of the sensors relative to the body [75].

Interference by external magnetic fields of the earth and the urban environment are typically avoided by enclosing the measurement setup in a magnetically shielded room. Inside a shielded room, a wearable sensor vest allows for free movement of the patient and, therefore, improved diagnostics of coronary artery disease by examination of the heart during exercise. For unshielded environments in the ward or at home other interference elimination approaches should be investigated further for use with a wearable vest. A common approach is to measure the gradient of the magnetic field with gradiometers, thereby eliminating most external interference [76,77]. Another approach is to measure the external interferences with one or several reference sensors and to subtract it [78]. During signal processing, residual interferences may be further eliminated by spectral filtering [79], averaging of heartbeats [32], signal decomposition approaches, and common mode rejection.

Prospectively, a magnetic sensor vest could be fitted with electrodes in order to simultaneously record MCG and ECG signals. The electrode positions could be optimized together with the magnetic sensor positions and orientations in order to maximize the yield of complementary information about the common sources. Currently existing electrode vests primarily aim at sampling the whole torso evenly and have yet to be validated for clinical applications [80].

## 5. Conclusions

We conclude that preferential locations and orientations of magnetic sensors can be derived. The novel optimization of sensor orientations identified magnetic field components tangential to the body surface as important. Optimized whole torso coverage can significantly improve the condition of the corresponding linear inverse problem. A wearable magnetic sensor vest utilizing magnetoresistive or optically pumped magnetometer technology is practical for non-invasive clinical diagnostics in cardiology. The optimized sensor arrangement could facilitate the definition of a robust standard for sensor placement in magnetocardiography.

## Figures and Tables

**Table 1:** Statistics of individual optimized setups and cluster-based representative setups. Values are the mean ± standard deviation unless marked otherwise.

	*CN*	*Skeel*	ρ
Value of randomly initialized vest setups	209 ± 88	159 ± 50	5.48 ± 0.64
Value of optimized vest setups	19.05 ± 1.15	26.21 ± 1.21	2.51 ± 0.05
Number of sensors at edge of vest	47.2%	46.9%	40.3%
Median distance to cluster medoid	30.8 mm	27.9 mm	29.6 mm
Maximal distance to cluster medoid	108.1 mm	110.8 mm	131.3 mm
Shape of orientation distribution per cluster	1.76 ± 1.80	1.45 ± 0.84	1.91 ± 2.31
Strength of principal orientation per cluster	2.08 ± 0.84	2.84 ± 0.84	1.99 ± 0.77

**Table 2:** Goal function values of cluster-based representative setups.

		*Evaluated with:*		
		*CN*	*Skeel*	ρ
Cluster-based representative setup derived using:	*CN*	**49.49**	56.79	3.41
	*Skeel*	133.97	**99.39**	3.29
	ρ	106.90	85.01	**3.49**

## Abbreviations

The following abbreviations are used in this manuscript:

AV	Atrioventricular
BSPM	Body surface potential map
CN	Condition number
ECG	Electrocardiography
MCG	Magnetocardiography
MR	Magnetoresistive
OPM	Optically pumped magnetometer
PAM	Partitioning around medoids
PSO	Particle swarm optimization
SNR	Signal-to-noise ratio
SQUID	Superconducting quantum interference device
